# The Feasibility and Usability of an Artificial Intelligence-Enabled Conversational Agent in Virtual Reality for Patients with Alcohol-Associated Cirrhosis: A Multi-Methods Study

**DOI:** 10.1089/jmxr.2024.0033

**Published:** 2024-11-11

**Authors:** Yee Hui Yeo, Allistair Clark, Muskaan Mehra, Itai Danovitch, Karen Osilla, Ju Dong Yang, Alexander Kuo, Hyun-Seok Kim, Aarshi Vipani, Yun Wang, Walid Ayoub, Hirsh Trivedi, Jamil S. Samaan, Tiffany Wu, Vijay H. Shah, Omer Liran, Brennan Spiegel

**Affiliations:** 1 Department of Medicine, Cedars-Sinai Medical Center, Cedars-Sinai Center for Outcomes Research and Education (CS-CORE), Los Angeles, California, USA.; 2 Karsh Division of Gastroenterology and Hepatology, Department of Medicine, Cedars-Sinai Medical Center, Los Angeles, California, USA.; 3 Department of Psychiatry and Behavioral Neurosciences, Cedars-Sinai Medical Center, Los Angeles, California, USA.; 4 Department of Psychiatry and Behavioral Sciences, Stanford University School of Medicine, Palo Alto, California, USA.; 5 Comprehensive Transplant Center, Cedars-Sinai Medical Center, Los Angeles, California, USA.; 6 Cedars-Sinai Medical Center, Samuel Oschin Comprehensive Cancer Institute, Los Angeles, California, USA.; 7 Division of Gastroenterology and Hepatology, Mayo Clinic, Rochester, Minnesota, USA.

**Keywords:** motivational interviewing, accessibility, immersive environment, nonjudgmental, thematic analysis

## Abstract

**Background & Aims:**

Patients with alcohol-associated cirrhosis often lack access to effective interventions to prevent drinking. We assessed the feasibility and usability of an artificial intelligence (AI)-enabled conversational agent in virtual reality to deliver self-administered mental health support.

**Methods:**

Our multidisciplinary team combined spatial computing and AI to deliver an immersive form of cognitive behavioral therapy and motivational interviewing using established relapse prevention protocols. Participants engaged with the program privately for 30 min, followed by a semistructured interview. Usability was measured with the intervention usability scale (IUS). Thematic content analysis of interviews was conducted to extract themes and insights.

**Results:**

Of 20 participants, 50% reported active alcohol use. The median Model for End-Stage Liver Disease 3.0 score was 22. All participants used the full 30 min of psychotherapy; no severe adverse events were reported. The IUS score was acceptable (≥75) in 78% of participants. Thematic analysis distilled 509 first-level codes into nine overarching themes. The program quickly established rapport, providing positive reinforcement and actionable goals perceived as nonjudgmental. About 80% felt themselves open up to the avatar; 85% reported the experience as beneficial and noted belief in potential efficacy. Many endorsed the privacy, accessibility, and immersion of the program. While a minority preferred human therapists and noted shortcomings in the program, 90% expressed interest in further use.

**Conclusions:**

Combining spatial computing and AI is perceived to be a safe, acceptable, easy-to-use, nonjudgmental, and immersive form of self-administered psychotherapy among patients with alcohol-associated cirrhosis. Studies to examine the impact on cessation are needed.

## Introduction

Alcohol consumption significantly impacts global health, is attributed as a causal factor in over 200 disease and injury conditions, and is ranked as the fourth leading preventable cause of death worldwide.^1,2^ It leads to approximately 3 million annual deaths, accounting for 5.3% of all global mortality.^3^ Notably, in the United States, over 29 million individuals were identified with alcohol use disorder (AUD) in 2019, with an observed increase in related mortality during the COVID-19 pandemic.^4,5^ Alcohol-associated cirrhosis, a severe complication of AUD, carries a dire prognosis with a significant reduction in life expectancy.^6^ Global per-capita alcohol consumption increased from 1990 to 2017 and is projected to increase further by 2030.^7^ Despite this, treatment access is low, with only 7.6% of affected individuals receiving AUD-related treatment in 2021.^8^ Furthermore, only 16% of individuals receiving AUD-directed therapy achieved abstinence.^9^

Treatment modalities for AUD encompass both pharmacotherapy and psychotherapeutic approaches, with increasing emphasis on combination therapy.^10,11^ Psychotherapeutic approaches, such as motivational interviewing (MI) and cognitive behavioral therapy (CBT), are widely disseminated evidence-based interventions for AUD and recommended by professional societies as they serve to address harmful drinking patterns.^12,13^ Additionally, psychotherapy is linked to a lower risk of alcohol-associated liver disease progression in AUD patients and reduced decompensation risk in those with alcohol-associated cirrhosis.^14^ However, relapse prevention treatment remains a challenge, with more than 60% relapsing within the first year of treatment.^15,16^ Furthermore, limited treatment access is aggravated by a scarcity of mental health professionals, pervasive societal stigma, financial, attitudinal, and geographic barriers.^17,18^

Technological solutions, such as telemedicine and mobile applications, have begun to address barriers to access.^19,20^ Among these advancements, virtual reality (VR) is emerging as a valuable supplementary tool for AUD assessment and treatment.^21^ VR shows potential in reducing cravings through cue-exposure therapy (CET)^22^ and in administering avoidance training for alcohol dependence.^23^ Ongoing studies are investigating the efficacy of VR-assisted CBT in augmenting traditional CBT.^24^ However, VR interventions, thus far, have involved preprogrammed or scripted methods. Recently, advancements in artificial intelligence (AI) have introduced large language models (LLMs) capable of generating more dynamic and responsive interactions. LLMs have shown a remarkable ability to facilitate personalized treatment plans and enhance patient engagement in the fields of medicine.^25^ The applications of VR with integrative, interview-based therapies enabled through LLMs remain unexplored.

Our study introduces a novel program that combines spatial computing with AI to deliver self-administered mental health support for patients with AUD. In earlier research, we found this program to be feasible, safe, and acceptable for patients experiencing mild-to-moderate depression or anxiety.^26^ Building on this foundation, we aimed to adapt the program to integrate MI techniques, as well as to assess its feasibility, safety, and efficacy in delivering combined CBT and MI in VR to address persisting gaps in psychotherapeutic care delivery for patients with alcohol-associated cirrhosis.

## Methods

This qualitative study was approved by the institutional review committee at Cedars-Sinai Medical Center (IRB STUDY2753). GPT-4 (OpenAI) was utilized to improve the language and readability of the first draft. All outputs from GPT-4 were reviewed and edited by the authors. All authors are fully responsible for the content of the article.

### Description of intervention

We previously developed an AI-enabled, self-administered mental health support tool in VR environments for those with anxiety and depression at Cedars-Sinai Medical Center in Los Angeles, California.^26^ Using a modified LLM (GPT-4, OpenAI), the program was designed to comprehend patients’ inquiries, ask follow-up questions for additional medical history, and respond at a comprehensible level.

**FIG. 1.**
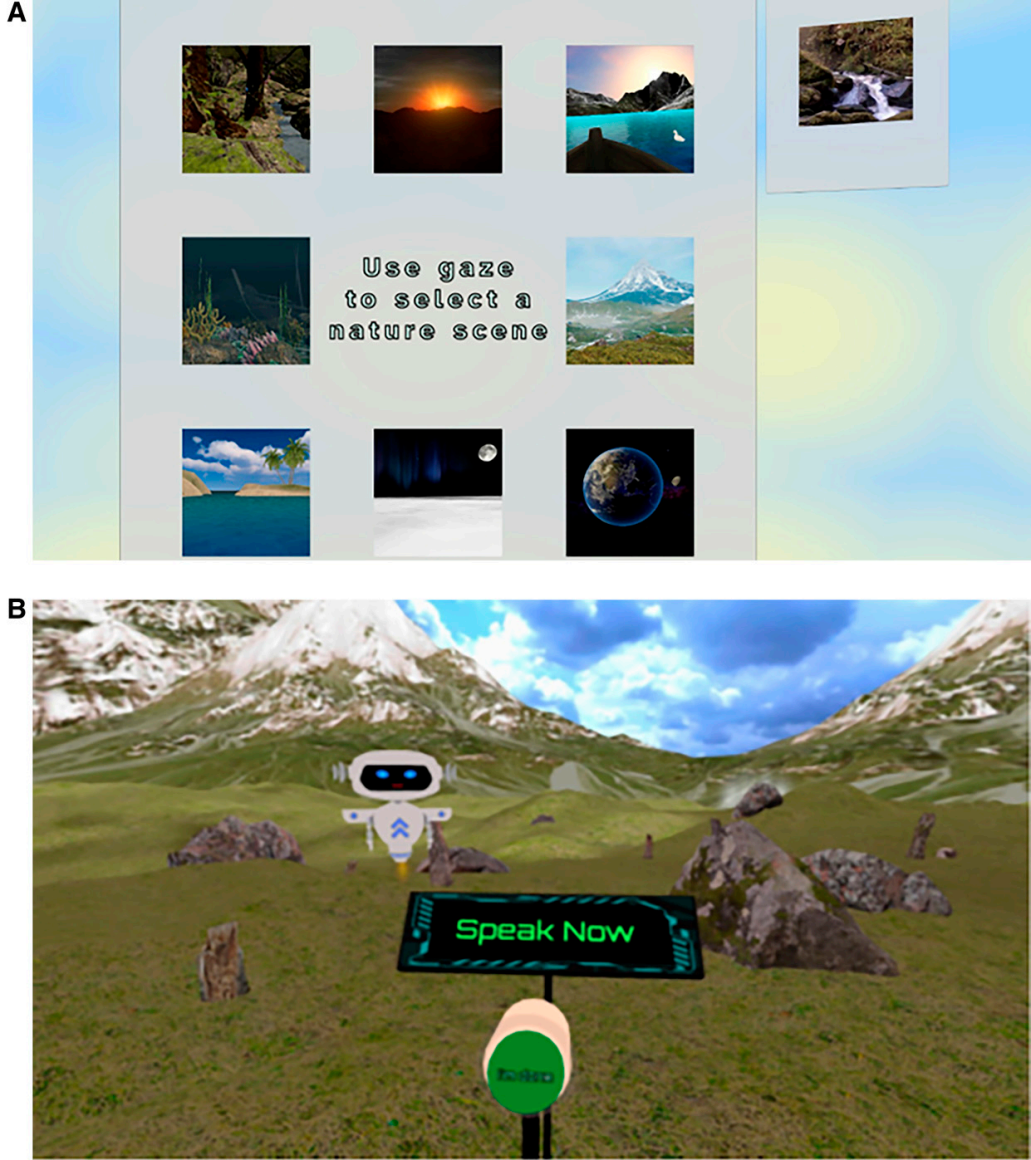
Virtual reality (VR) environment and user interface. **(A)** Nine distinct VR scenes designed for immersive psychotherapy. **(B)** The conversation setting within the VR environment. Participants can begin speaking when the “Speak Now” light is on. After speaking, they can conclude by selecting the green “I’m done” button.

Upon program launch, users can select from nine immersive nature scenes (Fig. 1A), including aquatic settings (tropical beach, glacial lake, coral reef), terrestrial settings (desert sunrise, snowy mountain, verdant forest), and celestial settings (floating above the clouds, orbiting Earth). Within these environments, the user is greeted by “Xaia,” a conversational avatar embodied as a robot that serves as the therapist (Fig. 1B). Once the participant converses with the avatar, the backend system securely transmits audio recordings to a Health Insurance Portability and Accountability Act (HIPAA)-compliant server. These recordings are converted to text using speech-to-text AI (Whisper, OpenAI). The text is then processed by the trained and fine-tuned LLM to generate a response, which is converted back to a speech by a text-to-speech AI (ElevenLabs Inc.) trained in a calming and reassuring tone. This final output is uploaded to the VR headset and delivered to the user, enabling continual conversation with the avatar. Once the conversation concludes at the discretion of the user, the program summarizes the dialogue and closes the session. For a video demonstration of a user interacting with the avatar, visit this link: https://virtualmedicine.org/XAIA/paper/video.

### Integration of MI into the program

To incorporate both CBT and MI techniques into the program, a psychiatrist specializing in addiction medicine (I.D.) and a psychologist (K.O.) with extensive experience in MI served as standardized patients, providing valuable feedback to refine the avatar’s capabilities. After many iterative cycles of feedback, refinement, and testing, the AI therapist increasingly incorporated techniques associated with MI and CBT.

### Participant recruitment

We aimed to recruit up to 20 participants, 18 years of age or older, via a combination of convenience and snowball sampling methods. Participants were required to speak English and have a diagnosis of alcohol-associated liver cirrhosis from the Cedars-Sinai hepatology outpatient or inpatient service. We excluded individuals with a history of severe motion sickness, facial or head deformities, seizures in the past year, current pregnancy, or legal deafness or blindness. We intended to halt recruitment once our analyses indicated that thematic saturation was reached.

### Study procedures and data collection

To evaluate how well the conversational avatar could provide key psychotherapeutic techniques, including CBT and MI, while fostering an empathetic and supportive therapeutic relationship, a 30-min interview session was offered to the participants. Consenting outpatients and inpatients (in their hospital room) participated in a single therapy session facilitated by study staff. Participants were initially briefed on the use of the headset (Meta Quest 2, Meno Park, CA) and filled out demographics, Generalized Anxiety Disorder (GAD)-7, Patient Health Questionnaire (PHQ)-9, and Alcohol Use Disorders Identification Test (AUDIT) questionnaires before engaging privately with the conversational avatar for up to 30 min, at which time staff ended the session. GAD-7 is a screening tool for anxiety disorder. A GAD-7 score of ≥9 demonstrated high sensitivity (80%) and specificity (86%) for identifying any anxiety disorder in patients with AUD.^27^ PHQ-9 ≥12 had a sensitivity of 81% to identify depression in individuals with drug or alcohol addiction.^28^ AUDIT is a widely tested instrument for screening for alcohol misuse.^29^ A licensed mental health professional (O.L. or R.C.) remained nearby in case of an emergency. Participants then engaged in a 45-min, one-on-one, semistructured debriefing interview led by a trained qualitative researcher (A.C.), which was recorded and transcribed. The interviews aimed to collect feedback, experiences, and opinions about the session. Supplementary Data S1displays the interview guide.

### Intervention Usability Scale

After their 30-min interaction with the avatar, participants were administered the Intervention Usability Scale (IUS) to gauge their perceptions of the program’s usability (i.e., ease of use and enjoyment of use). The IUS is an adapted version of the systems usability scale (SUS), which has been modified to assess the usability of complex health interventions.^30^ The IUS has shown strong similarities to the SUS and has demonstrated acceptable validity and reliability (α = 0.83) evidence in past literature. An IUS score of 75 or above indicates adequate usability.

### Bracketing statement

We (A.C., M.M., and Y.H.Y.) operated as the main data collection, analysis, and authors of this article. As the software at the center of this investigation was developed and publicized by the lab that we work at and, in our respective ways, had a part in developing, we acknowledged at the start of trial operations that we would each have a level of implicit bias toward the success of conversational avatar within this research context. Consequently, we sought feedback from noninvolved lab members when creating the interview protocol and ensured that two investigators were present during all interviews to monitor for language that may bias the participant to respond in a way that confirmed or supported our preconceptions. Furthermore, the first analytical passes on initial transcripts involved repeated sessions of code consensus checking between all three of us. Finally, when writing the article and the results section, authors who were not involved in the analysis were encouraged to review the finalized result section extensively.

### Analysis

Three qualitative researchers (A.C., M.M., and Y.H.Y.) lead a qualitative content analysis to derive meaningful categories of participants’ experiences with the conversation agent from the transcribed interviews. They created codes and labels from the unstructured data through iterative passes, with each subsequent pass refining and aggregating the codes into categories supported by direct quotations from the transcripts. This approach involved three formal passes across the data.

In the first pass, raw data were approached using an open, or initial, coding process.^31,32^ Open coding allowed the researchers to approach the raw data in a more granular fashion while still letting new concepts and topics emerge with little interpretation on the part of the researchers or obfuscation of said data’s meaning. Within the open coding framework, two sub-methods were utilized in assigning and identifying nascent codes: *in vivo* coding and holistic coding. Regarding *in vivo* coding, this approach involved creating first-level codes that were direct quotations of impactful or significantly insightful statements made by participants during the interview. This aided in identifying supporting quotes, as used later in this article.

Holistic coding was utilized when large statements or passages created by the participants yielded significant meaning on multiple subtopics within a topic. The resulting holistic codes that were created were not direct quotations but rather refined descriptions of the participants’ statements (e.g., the passage “I just kept looking around at the water and just really put me at ease, like I didn’t realize how much the visual parameters allows my body to relax.” resulted in a holistic code of “VR Environment: relaxation”). Utilizing these two approaches within the open coding framework yielded a rich description of the data that provided a large corpus of first-level codes.

First-level codes were then refined and assimilated into a smaller number of broader second-level codes during the second passthrough of the data. This passthrough utilized a pattern coding approach, wherein similarities in subject and meaning between first-level codes were analyzed and codes that were shown to have high commonality were grouped into second-level codes. These second-level pattern codes reflected larger concepts or subcategories that emerged from the data that still had commonality between pattern codes (e.g., two pattern codes developed included “Easy to talk to” and “Understanding with listening skills” when describing the conversational avatar).

The third passthrough analyzed these second-level pattern codes through a focused coding approach. This approach entailed the selective grouping and categorization of the developed second-level pattern codes into an even smaller number of mutually exclusive code categories (using the pattern codes described above, “Easy to talk to” and “Understanding with listening skills” were eventually grouped under the theme “Overall Experiences of Therapy with the Conversational Agent”). These categories, being the highest level of refined data, described broad reoccurring concepts that existed across a majority of the data that overall give a clear depiction of participants’ thoughts, experiences, and opinions about the developed software.

This process occurred after each visit and continued until no new codes were identified, thus indicating thematic saturation. Within this investigation, saturation was identified via the emergence rate of new second-level pattern codes.

## Results

Twenty participants were required to reach thematic saturation. Table 1 displays the participants’ demographics. Participants were 46 years old on average (range 29–66), with 13 males (65%) and 7 females (35%). Thirty-five percent were Hispanic, and 45% were non-Hispanic White. The median (interquartile range) of the GAD-7, PHQ-9, and AUDIT scores were 6.5 (8.0), 7.5 (9.5), and 11 (20), respectively. Fifty percent had active alcohol use (last use of alcohol within the past three months). The median Model for End-Stage Liver Disease 3.0 score of the participants was 22. There were 30% of participants (6 out of 20) recruited from the inpatient service, and the rest were from outpatient.

**Table 1.** Participant Demographics

Variable	Number of participants	%
Overall	20	100
Age (mean, SD^a^)	19	46.1 (10.7)
Gender		
Female	7	35
Male	13	65
Race/Ethnicity		
White (Non-Hispanic)	9	45
Black or African American	1	5
Asian	3	15
Hispanic	6	30
American Indian or Alaskan Native	1	5
Highest level of education		
8^th^ grade or less	0	0
Some high school	1	5
High school graduate or equivalent	5	25
Some college	4	20
College degree	6	30
Advanced graduate degree	4	20
Combined household income		
Less than $10,000	2	10
Between $10–20,000	3	15
Between $20–50,000	2	10
Between $50–100,000	3	5
Between $100–200,000	6	30
More than $200,00	2	10
Prefer not to answer	4	20
VR experience		
No experience	6	30
A little bit of experience	9	45
Some experience	2	10
Quite a bit of experience	2	10
A lot of experience	1	5

a SD, standard deviation; VR, virtual reality.

No participants experienced any severe adverse safety events. No therapy sessions required psychiatric or gastroenterology/hepatology intervention by professionals. Two patients exhibited grade 1 hepatic encephalopathy before the start of the session; however, there was no exacerbation of their condition, and they successfully tolerated the therapy session. No incidents of motion sickness or related symptoms were reported. One patient noted a transient headache but managed to complete the 30-min session, with the headache self-resolving post-session.

### IUS

**FIG. 2.**
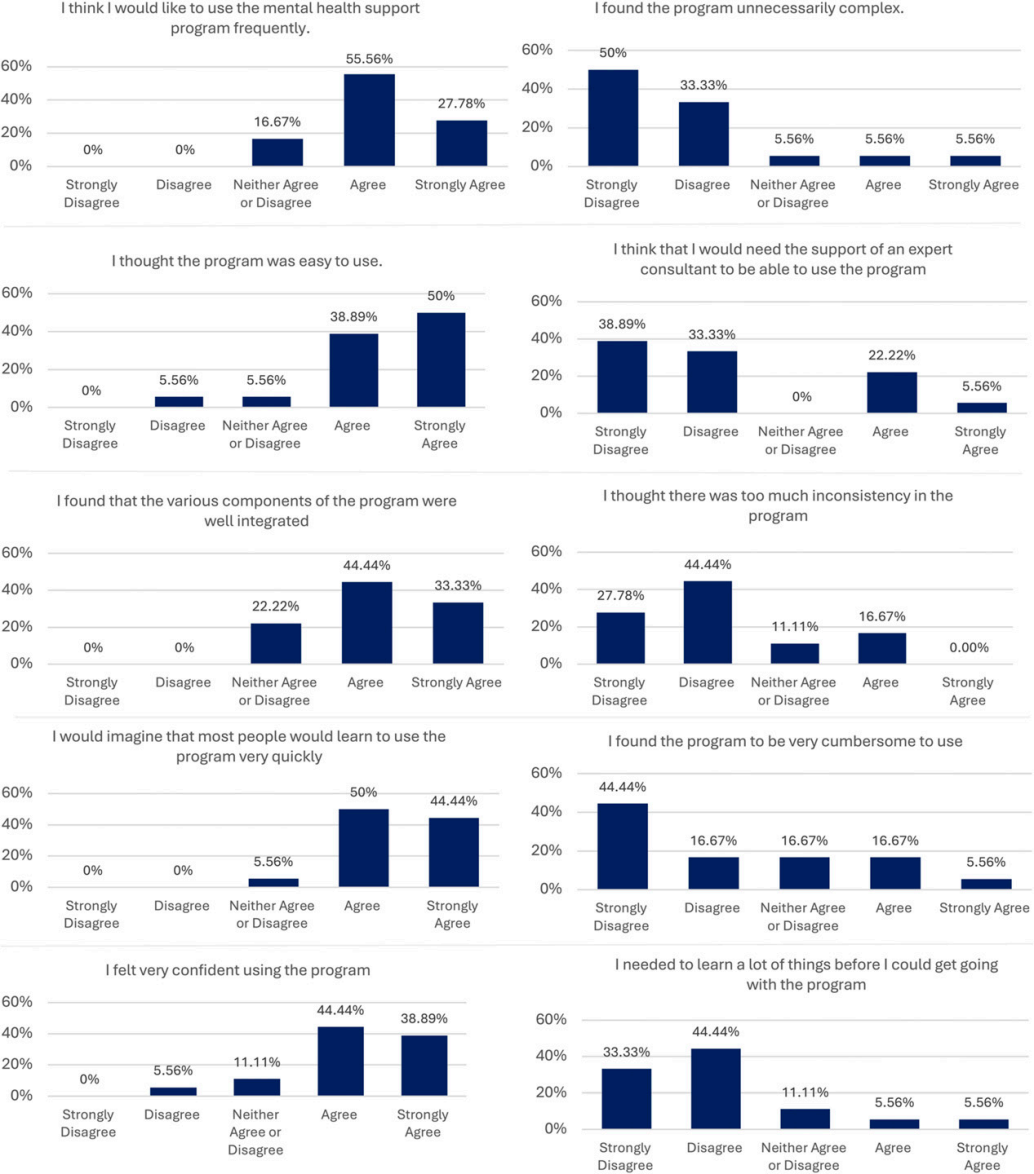
Bar chart showing the distribution of response category frequencies across each item of the Intervention Usability Scale (IUS) (as percentages). Item response options ranged from strongly disagree to strongly agree on a 5-point Likert Scale.

Overall, the majority of participants reported high IUS scores, with 14 out of 18 (78%) participants (excluding two participants with incomplete data) scoring ≥75. Figure 2 shows the distribution of responses per each question as percentages. Specifically, for the question “I thought it was easy to use,” 16 out of 18 (88.9%) participants agreed or strongly agreed. For the question “I would imagine that most people would learn to use the program very quickly,” 17 out of 18 (94.4%) participants agreed or strongly agreed. For the question “I think that I would need the support of an expert consultant to be able to use the program,” 13 out of 18 (72.2%) participants disagreed or strongly disagreed, while the remaining participants agreed or strongly agreed.

### Thematic content analysis

**FIG. 3.**
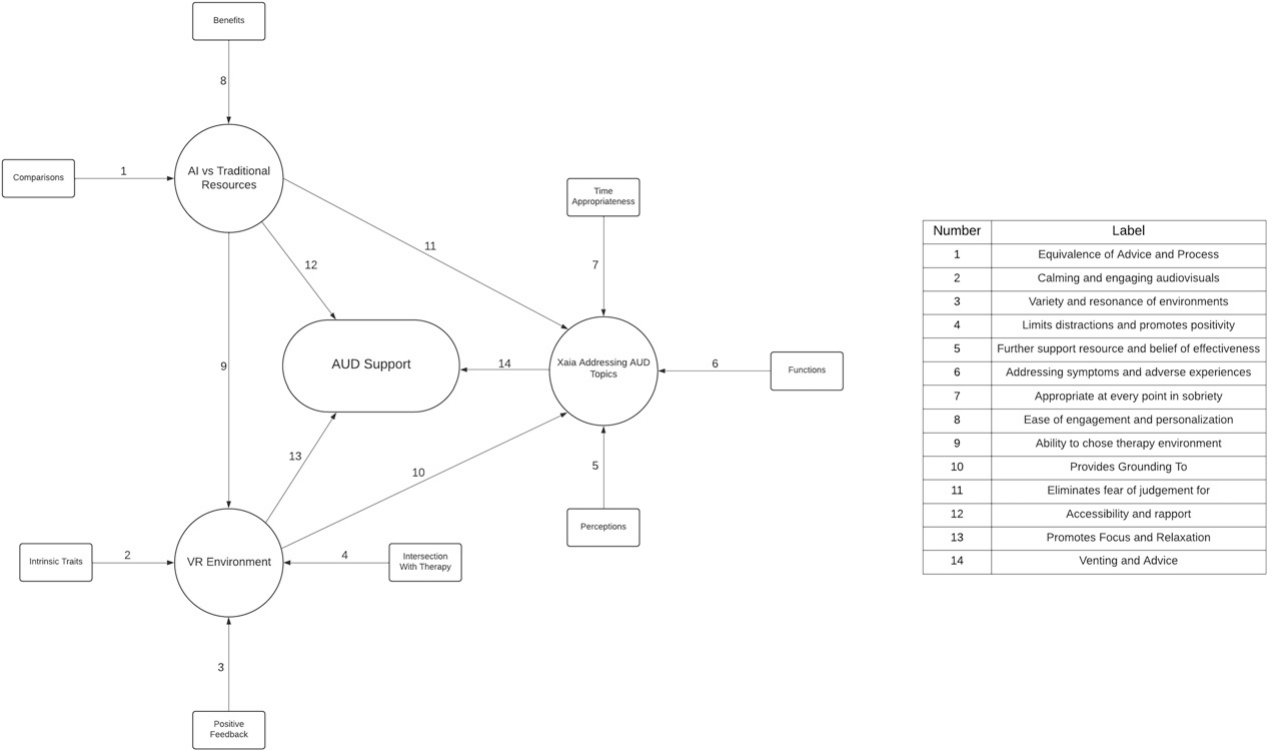
Web diagram illustrating how participants believe the program delivers support for alcohol use disorder (AUD) as derived from the thematic analysis. From the outside in, squares represent subthemes identified within each thematic category (circles). The ellipse in the center represents the general construct of providing support for alcohol use disorder. Arrows represent relationships between subthemes, themes, and AUD support. Arrows from subthemes to themes: (1) Equivalence of advice and process; (2) Calming and engaging audiovisuals; (3) Variety and resonance of environments; (4) Limits distractions and promotes positivity; (5) Further support resource and belief of effectiveness; (6) Addressing symptoms and adverse experiences; (7) Appropriate at every point in sobriety; (8) Ease of engagement and personalization. Arrows between themes: (9) Ability to choose therapy environment; (10) Provides grounding to; (11) Eliminates fear of judgment for [addressing AUD]. Arrows from themes to AUD support: (12) Accessibility and rapport; (13) Promotes focus and relaxation; and (14) Venting and advice.

First-pass coding yielded 509 first-level codes, which were refined and combined into 325. Further passes and refinement resulted in 35 second-level codes, categorized into nine themes discussed below (see Fig. 3 for the conceptualization of how participants reported the conversational avatar to provide AUD support). These themes include patient experiences prior to their session with VR, general descriptions of the program (appearance of the avatar, interactions with the avatar, and functions of the program), the program’s perceived effectiveness for AUD (AI vs traditional resources, perceived benefits for AUD), and final thoughts from the participants (VR environment, overall experience, and drawbacks and suitability). Table 2 exemplifies these themes with respective quotations.

**Table 2.** Illustrative Quotations by Theme

Theme/subtheme	Illustrative quotations
Prior experiences with therapy	**Stigmatized:** “if you talk to a psychiatrist or doctor, there’s something wrong with you and you don’t want to have nothing wrong with you” **Challenge in getting support:** “the last couple of years I have been in and out of rehabs and counseling sessions, hundreds of different sessions and counselors…it was an absolute failure for me.”
Descriptions of the program	
*Appearance of the avatar*	“simple robot with no expression lets you be and lets you talk.” and “a feeling of anonymity, yet openness.”
*Interactions with the avatar*	**Openness and engagement: “**once I started opening up to her… I felt like I could talk to this thing” and “as time went by it was easier for me to just talk.” “It was easier to open up to than my AA meetings,” and “I was brutally honest. I was crying…I feel lighter right now.” **Nonjudgmental:** “it was so easy just to say what I wanted to say and not worry about judgment,” and “It was just consistent… no judgment or anything. It felt just sort of natural.” **Personification of the avatar:** “We got to know each other a little bit… as far as like a therapist-client relationship would go.” and “It made me feel like she was actually listening… not something you get often from a conversation with a human being.”
*Functions of the program*	**Formulating coping mechanisms**: “She gave good suggestions, kind of like if I asked her something, she picked out specifics, which may alleviate time on reading all the different articles.” **Facilitating self-reflection:** “I’ve heard this a hundred times, but I’ve never put it into practice… and today I did.” **Bring up important subjects**: “if I tell her about my baby, she remembers and brings it up later, which makes the conversation feel more personalized.”
Perceived effectiveness for AUD	
*AI vs traditional resources*	**Easy communication:** “I think it’s easier than an in-person encounter.” **Remarkable accessibility: “**If you get something virtual like that, you can go back to it anytime, whereas therapists… you’re around their schedule.” and “This could be used for people in certain areas where they can’t afford the help.” **Providing secure environment:** “I felt safe knowing that she’s not going to go outside the room and discuss me with other people.” **Patient-driven:** “It ended when I was ready to end, not when the therapist was ready to end.”
*Perceived benefits for AUD*	**Environment to discuss sensitive issues: “**It takes a certain type of atmosphere to be able to release that stuff… this could be really useful for people who have suffered some serious traumatic stuff in their life.” **Understanding approach:** “She did a really good job of understanding what I was talking about and presenting relevant ways I could use tools to respond to those triggers.” **Personal feedback and positive reinforcement: “**Being able to talk about it and get those suggestions and that affirmation that you’re doing the right thing. I think it’s really important.”
Perception of the program	
*VR environment*	**Immersive effects:** “your mind gets distracted, and it opens up, instead of, you know, being in a real-world situation, you could just… be in the, in the VR and have a different experience.” **Less distractive:** “Um, and one of them I went to… it was on Beverly Boulevard and… it’s like the blur of the cars and the noise and there were times where it wasn’t as therapeutic as I was hoping. So… It’s quiet and it’s secluded and it’s calm and I feel pretty safe right now.” **Resonance with certain environments:** “I was in space so I felt like I was in my own safe little box” and “I’m an avid camper, okay, so… being outside, it’s cold, I’m on the water, uh, and then it’s, it’s walking me through the breathing. That was… top 10. Phenomenal.” **Audio effects:** “I like the sound, I like the waterfall and stuff.” and “Yes. Having sort of the calming sounds… You know, water trickling and things like that. Having that in the background, definitely.” **Preference for VR methods:** “Um, you’re not going to get that immersion… I think. Um, yeah, a 2D screen, you’re just, I don’t think you’re going to get that, uh, this is my safe place type of feeling.” and “I mean, I don’t think there’s any better way to present it these days. I certainly wouldn’t want to just look at a laptop screen, for instance.”
*User experience*	**Overall:** “*absolutely spectacular and really well done.”* and “I didn’t know they could build an AI that was even this effective. So, I’m honestly shocked.” **Relaxing and comfortable:** “I almost feel like I just, you know, went through a 30-minute meditation” and “a space to speak out with someone out loud, it just, it gave out my voice.” **Continue to use the program:** “if I were able to carve out the time, which I think I could more. I think I could do better at home. I do because I think it’d be a nice respite [from] the frustrations and the worry.” and “I kind of wanted to start over and go down some other area, you know, so that I could get more out of it. You know, out of the interaction.”
*Drawbacks and suitability*	**Tone and appearance:** “… a bit too happy” and “a little girlish sounding… a little too young” and “like Clippy from Microsoft Word.” **Advice quality:** “lack of insight or applicability” and “her answers to my problems were kind of just generic answers that you would get from any therapist” and “I felt like some of the questions she was asking felt repetitive. It almost felt like you’re reading from a book.” **Lack of human connection and accountability:** “But with a human, it’s like… a hug. Like a hug, like you know, after your sessions and stuff, they let you know that everything’s going to be okay,” and “In person therapy, you have a specific appointment time. and so you have this accountability, so it would [have to] be my own accountability.”

AI, artificial intelligence; AUD, alcohol use disorder; VR, virtual reality; AA, alcoholics anonymous.

### Prior experiences with therapy

Participants were asked to share their prior experiences with therapy, if applicable. They mentioned the stigma associated with receiving help. Some participants conveyed that they sometimes felt judgment or inauthentic compassion with an in-person therapist. One patient reported the sheer exhaustion of trying to receive the right support. Participants were also asked about their prior experiences with technologies such as VR and AI, and they had mixed levels of familiarity. Only a few participants were very familiar with VR and owned their own headsets. Most knew of VR and its “*commercial fun uses*” from media sources like “*TikTok and Instagram*” but had little to no experience with using VR nor had seen its use for health-related applications. These participants expressed that due to the novelty of the program, they would need time to “*process*” what they experienced and “*get used to*” it. Alternatively, participants who were more tech-savvy identified technical limitations that shaped their impressions of the program.

### Physical characteristics of conversational avatar

Notably, some of the first comments on the program focused on the physical characteristics of the embodied avatar, revealing several insights into the users’ perceptions and reactions. While some participants recognized that they were “*talking to a robot*,” they found the nonhuman features less distracting and misleading than if the avatar was “*pretend[ing] to be like a human being*.” The minimalist anthropomorphic appearance, featuring “*a face, eyes you could look into, and then a mouth*,” was remarked as simple and effective in focusing the dialogue. This suggests that the design of the avatar facilitated a comfortable space for open dialogue.

### Interactions with the conversational avatar

The interactions between the avatar and several participants revealed a dynamic evolution from initial curiosity and skepticism to increased engagement and comfort. Initially, many felt “*closed off*” and “*kind of weird talking to a robot*.” However, as conversations progressed, this perception shifted dramatically, and they felt “*completely open and honest*.” Eighty percent of participants stated feeling that they were opening up to the avatar. The participants described that they began to “*trust*” the avatar over time. Moreover, the avatar’s ability to recognize personal details like names helped establish a quick rapport.

Many participants emphasized the nonjudgmental feedback and unique openness that allowed users to share deep personal thoughts they might typically withhold, such as some of the “*more awful things that happened to [them] during [their] addiction.*” Several mentioned that they would want to engage in more sessions with the avatar to “*know [them] better and provide better suggestions*.” The interviews also revealed insights into patient perceptions of the avatar as “*empathetic*” and “*caring*.” They commented that its approach made them feel “*supported*” and “*like she had* [their] *back*” without feeling “*talked down to or anything.*” A few participants also began to blur the distinction between robot versus in-person interactions. “*We got to know each other a little bit … as far as like a therapist-client relationship would go*.”

### Functions of conversational avatar

The avatar was noted to provide actionable goals and salient advice. Its responses were recognized by participants to be not only intuitive but also specifically tailored. It helped participants formulate coping mechanisms, implement restructuring techniques that addressed underlying cognitive distortions, and facilitate self-reflection. Participants were particularly impressed with the avatar’s approach to offering new perspectives and methods they had not previously considered. Moreover, the avatar was noted for being quickly responsive to identified problems and sensitive to triggers. This responsiveness extended to its ability to conduct in-depth discussions on sensitive topics. Participants felt that the therapy was participant-led, with the avatar facilitating a conversation where they could “*dive deeper*” into personal issues. Communications were consistently described as calming and natural. This ease of communication was underscored by the avatar’s ability to recall and bring up important past subjects.

### AI vs. traditional resources

Participants noted several distinctions and advantages associated with using an AI conversational avatar compared to conventional therapy and support groups like Alcoholics Anonymous (AA). One participant stated how “*it’s completely different … It made me feel good*” and highlighted the less intrusive nature of the virtual interaction. Another noted the ease of interaction with the avatar. It was also indicated that the avatar provided equivalent quality advice to traditional therapy, with added benefits like constant accessibility and no judgment. One said, “*It made me feel like it was specific information that was useful to me*,” drawing a parallel between the advice given by the AI avatar and that of human therapists. Another participant pointed out the advantages of AI in emergency or high-need contexts.

Others commented on its round-the-clock availability anywhere and its ability to overcome geographical barriers to access. Notably, this AI therapy was repeatedly remarked for providing a safe environment. The patient-driven pace of therapy also allowed for more autonomy and control over sessions. This statement illuminated the patient-centered nature of AI therapy that conventional therapeutic encounters may lack.

### Perceived benefits for AUD

Participant responses explored the avatar’s capabilities in addressing addiction-related challenges. They recognized its ability to foster an environment conducive to discussing sensitive issues such as addiction-related trauma and guilt. A participant appreciated the avatar’s direct but supportive feedback on steps toward recovery, saying the responses were “*very affirmative … but a little more in-depth than that*.”

Importantly, the avatar was effective in identifying triggers and offering strategies to manage them, thus potentially avoiding relapse. Its approach was deemed as understanding and helpful. Furthermore, participants valued the positive space the program offered for discussing struggles and receiving encouragement. The program’s ability to work with specific symptoms of cirrhosis and provide personalized feedback was also noted to be more beneficial than conventional support systems.

### VR environment

Participants highlighted how the virtual setting and overall method of delivery of the program supported the therapeutic process. This included a phenomenon wherein the use of VR and being immersed in the virtual environment helped limit the distractions from their physical surroundings and “*… attracts your attention with the beautifulness of* [the VR Environment].” This immersive effect facilitated engagement in therapy. Some participants perceived the immersiveness to be less distracting than previous experiences with AA. Participants also found the virtual environments “*just calming and peaceful*” and enjoyed the “*assortment*” of environments “*that I could go into.*” Some participants indicated personal resonance with certain environments. Several also remarked on the positive benefits of the audio effects. Overall, the immersive quality of VR was considered by the participants’ superior to more standard digital delivery methods.

### Overall experience

When asked to reflect on their overall experience with the 30-min session, many participants reflected positively, with 85% noting belief in its potential efficacy. “*Excellent. Absolutely excellent,*” or that their time with the program was “… *pretty amazing*.” Others were “*… blown away by the technology*.” Participants often shared a similar sentiment in which they described themselves as impressed or shocked by what they just experienced.

Overall, 85% of participants reported the experience as beneficial. Participants elaborated further on their positive reviews, stating specific benefits such as relaxation or having a comfortable environment “*… because I felt like it was private, you know*.” Others remarked on it being “*a very calming experience*.”

Beyond this, 90% of participants indicated that they had continued interest in using the program again. Finally, when asked if they would recommend this program to other individuals they know who may face similar challenges, participants responded positively, “*especially some of the friends that I’ve made at AA*.”

### Drawbacks and suitability

Important shortcomings were also identified that should be considered for further development. For some participants, the tone and appearance of the robot avatar and the repetitive advice felt dissonant with the context of the session. Overall, this related to a feeling of a “*lack of guidance*” and a type of “*sterility*” among some participants, concerning both tones and content of the interaction.

When comparing the AI/VR program to traditional psychotherapeutic resources, participants noted a similar phenomenon to the sterility identified above. This absence of a human therapist was also noted to relate to a lack of potential follow-through or “*accountability*” in the sustained use of the program. Other concerns envisioned by participants included privacy concerns and the potential of “*overdependence*” on the program, a particular concern for a population with “*addictive tendencies*.”

## Discussion

Building upon the insights drawn from the thematic content analysis with 20 socioeconomically diverse participants, our study evaluated a program combining spatial computing and AI to deliver self-administered, immersive mental health support targeting alcohol craving reduction. The participants offered varied but mostly positive feedback on their interaction with the virtual AI therapist. They highlighted the program’s usability and acceptability, its personalized engagement, as well as the empathetic, private, and nonjudgmental therapeutic alliance it fostered. Participants described the AI avatar as realistic and engaging in its communication style, which many reported to be comparable with a human therapist. Most participants expressed a willingness to recommend this form of self-administered psychotherapy to others. These findings are notable given that three-quarters of participants reported minimal prior experience with VR.

The use of VR-enabled programs is known to facilitate CET,^21,33^ and there is burgeoning evidence suggesting that digital interventions can provide real-time support.^34,35^ Gamito et al. found how a therapist-guided, VR-based cognitive training intervention, compared to a control, improved the attention and cognitive flexibility of recovering AUD patients.^36^ Similarly, Langener et al. developed alcohol refusal training in VR with a preprogrammed peer pressure simulation.^37^ However, to the best of our knowledge, no existing studies have integrated real-time, dynamic, LLM-driven MI for craving reduction within a VR framework. MI is known for its nonjudgmental and nonconfrontational approach, which effectively enhances motivation to cease drinking by fostering a supportive therapeutic environment.^38^ Unlike prior studies that have explored VR or AI in isolation, our study is the first to combine these technologies to deliver a dynamic, immersive therapeutic experience for patients with cirrhosis. The combination of VR and AI-driven strategies could open new avenues for addressing AUD in a highly personalized and engaging manner.

The acceptable usability of the program, as indicated by high IUS scores, emphasizes its potential to achieve promising adherence.^30^ Furthermore, in our study, participants valued the immersive VR environment for its ability to separate them from external distractions. Unlike other audiovisual technologies, VR has a unique capacity to elicit profound emotional responses within a safe, therapeutic, virtual environment. The immersive effect of VR on its user is mediated through several mechanisms, most notably presence—the ability of VR to convey a compelling sense of “just being there,” wherever there happens to be. Relatedly, VR also has the unique ability to create a transitional space, a concept espoused by Winnicott, which is a therapeutic environment existing conceptually between the inner reality of the individual and the external world where creative processes and psychological growth occur.^39^ From a clinical standpoint, and as reported by the participants in this study, VR environments can provide a safe and controlled space for patients to explore and confront challenging situations, emotions, or memories in a supportive setting. By creating a transitional space, VR therapy encourages patients to focus their attention and engage actively with their experiences, facilitating emotional processing, behavioral change, and symptom reduction. This is reported to enhance focus and engagement during therapy sessions. Furthermore, the immersive aspect of VR is enhanced through the sense of presence.^40^ In VR settings, presence is defined as the sense of “being there,” where the individual feels fully immersed in the virtual environment as if they are physically situated within the simulated world. In our study, for example, one participant remarked, “*your mind gets distracted, and it opens up, instead of, you know, being in a real-world situation, you could just … be in the, in the VR and have a different experience*.” This reflects the core idea of presence, where the virtual world commands enough attention that the participant feels removed from the constraints of their physical environment. The combination of presence and the AI-driven interaction provided a unique therapeutic space that participants found more calming and less intimidating than traditional conventional settings, such as AA meetings or conventional therapist consultations. The nature scenes in VR contributed significantly to participants’ sense of relaxation and emotional well-being.^41^ These features may facilitate deeper emotional and therapeutic engagement.^42,43^ Future studies should compare the effectiveness of AI-driven therapy in immersive VR settings versus traditional app-based or web-based platforms to better understand the added value of VR immersion.

It is also important to note that about a quarter of the participants rated a need for an expert consultant to help them use the VR headset and the beginning of the program. In further analysis, we noted that the majority of participants who expressed a need for expert assistance were first-time users of VR technology. Their feedback indicated that the unfamiliarity with the VR headset, rather than the AI-driven avatar, was the primary factor contributing to their difficulty. These participants reported challenges such as adjusting the headset, navigating within the VR environment, and understanding how to start or end the session independently. Despite these initial usability challenges, the participants’ feedback about the conversational avatar itself remained highly positive (90% of participants indicated that they had continued interest in using the program again).

Despite these advantages, there were shortcomings of this approach identified by some participants. The character and tone of the avatar’s interaction, while generally well-received, were perceived by some as overly youthful or lacking in gravitas. Additionally, some participants noted the absence of human connection and the potential for reduced accountability. These insights suggest that while this approach may offer a novel avenue for treatment and support, it is not without challenges and may not be suitable for all people with alcohol-associated cirrhosis. The concerns regarding data privacy also underscore the need for careful consideration of ethical and practical implications. Additionally, selection bias could impact our results, as participants may have predispositions toward technological solutions. However, it is recognized that not all individuals with alcohol-associated cirrhosis prefer such interventions, with some favoring traditional, human-centric interactions given the proportion of individuals who have never experienced VR before. Despite this, the use of an AI avatar is not intended to replace but ideally to augment existing therapeutic options. Its utility, particularly for those who find it acceptable and beneficial, may be meaningful—especially given the current gaps in timely and effective treatment. Acknowledging the diversity in patient preferences, the introduction of an AI therapy avatar aims to enhance the spectrum of support available during the alcohol cessation journey. Furthermore, the findings obtained from 20 patients with only one-time use of the conversational avatar may not be conclusive. Future studies with a larger sample size and longitudinal use are warranted to evaluate the sustainability of engagement and the long-term efficacy of AI-driven interventions in managing AUDs and related conditions.

Future research should explore the integration of AI-driven VR therapies with traditional treatment modalities, assessing their combined effectiveness to maximize therapeutic outcomes compared to traditional modalities alone. Additionally, the content of MI or CBT delivered by the avatar was largely patient-driven. The avatar dynamically employed MI strategies when patient dialogue indicated ambivalence about alcohol use or motivation for change. In contrast, general CBT strategies, such as cognitive restructuring or coping skills training, were employed when participants expressed more concrete concerns, such as stress or anxiety triggers. Future studies are needed to determine the specific rates or proportions at which MI versus CBT strategies were used and whether these psychotherapeutic skills are practiced appropriately.

In conclusion, we have developed an AI-enabled conversational avatar that delivers self-administered mental health support for craving control within an immersive VR that is accessible and available without relying upon the availability of other mental health support resources. It is perceived to be usable, feasible, and safe. The personalized engagement and nonjudgmental positive reinforcement of this approach enhance the user experience, and several participants would like to recommend it to others.

## Abbreviations Used

AI: artificial intelligence
AUD: alcohol use disorder
CBT: cognitive behavioral therapy
CET: cue-exposure therapy
IUS: Intervention Usability Scale
LLM: large-language model
MI: motivational interviewing
SUS: systems usability scale
VR: virtual reality.
